# The practice environment’s influence on patient participation in intermediate healthcare services – the perspectives of patients, relatives and healthcare professionals

**DOI:** 10.1186/s12913-021-06175-z

**Published:** 2021-02-25

**Authors:** Linda Aimée Hartford Kvæl, Astrid Bergland

**Affiliations:** 1 Department of Physiotherapy, Faculty of Health Sciences, OsloMet - Oslo Metropolitan University, Oslo, Norway; 2 Norwegian Social Research (NOVA), OsloMet - Oslo Metropolitan University, Oslo, Norway

**Keywords:** Practice environment, Patient participation, Intermediate care, Older patients, Rehabilitation, Person-Centred care, Norway

## Abstract

**Background:**

Intermediate care (IC) bridges the clinical pathway of older patients transitioning from the hospital to home. Currently, there is a strong consensus that the practice environment is an important factor in helping older people overcome their limitations and regain function after illness or injury. Regardless of the arising attention related to person-centred care, the practice environment is yet to be recognised as a vital part of care, and a small extent of focus has been given the environmental dimensions of IC services. Thus, more research is required regarding the complex relationships between older people and the practice environment. This study explores the perspectives of older patients, their relatives and healthcare professionals related to the practice environment’s influence on patient participation among older people in the context of intermediate healthcare services.

**Methods:**

Using purposive sampling and theoretical approaches, including frameworks of patient participation, the practices environment and person-centred care, semi-structured interviews were conducted with 15 older patients, 12 relatives and 18 healthcare professionals from three different IC institutions in Norway to discuss their experiences and preferences regarding patient participation. A thematic analysis was used to explore patterns across the interviews.

**Results:**

Three main themes were identified: ‘location and access to physical facilities’, ‘symbolic expression of patients’ and professionals’ possibilities’ and ‘participating in meaningful activities’. The findings show that both the physical and the psychosocial environments influenced older patients’ various types of participation in IC services.

**Conclusions:**

To optimise rehabilitation care for older people, the ward configuration should focus on supportive environments that facilitate patient participation and provide options for the patients and relatives to independently access the facilities, balancing the personal capabilities with the environmental demands. To foster patient participation, the practice environment should thus align with the model of person-centred rehabilitation.

**Supplementary Information:**

The online version contains supplementary material available at 10.1186/s12913-021-06175-z.

## Background

Demographic changes, increased life expectancy, the political goal of reducing the duration of hospital stays and thus the fact that older persons are being discharged from hospitals both quicker and sicker than in the past are of great importance for health services [1]. Although older patients state that disabilities and reduced function are reasons for lack of participation [2], several studies have shown that external factors, such as physical, social and societal environments, also represent obstacles to older patients’ participation in healthcare services [3, 4]. Accordingly, in clinical patient pathways from hospital to home, quality collaborations between different levels of the healthcare system and the sharing of user-friendly information between patients, their relatives and healthcare professionals are important for older patients participating in their own care [5] along with supportive and stimulating healthcare environments that align with the model of person-centred rehabilitation [6].

In light of these demographic changes, intermediate care (IC) services aim to bridge the transition from hospital to home by delivering interdisciplinary rehabilitation for a short time after hospitalisation to prevent further hospital admissions [7]. The IC services can be provided in nearby municipal institutions, in nursing homes, in hospital units or in patients’ homes. Patient participation is a cornerstone of IC and consists of a patient’s rights and opportunities to influence and engage in the decision making about his care [8], which implies seeing the patient as a person, to pay attention to the importance of alliances that underpin the mutual engagement in activities within accommodating environmental configurations [9]. Hence, a pivotal understanding is that patients acquire more control over their own lives and choices of care through increased participation [10]. Nevertheless, research underlines that patient participation within IC services is a complex phenomenon that still remains outside mainstream practice and needs further investigation and exploration [2, 11, 12].

Currently, there is a strong consensus that the practice environment is an important factor in helping older people overcome their limitations and regain function after illness or injury. In general healthcare settings, it has been shown that activities and social interactions have a positive influence on older people’s health, well-being and quality of life [13, 14]. Ulrich [15] claimed that the practice environment in rehabilitation must go beyond costs and efficiency and must provide a stress-free atmosphere for healing, while Gesler [16] suggested categorising the ‘therapeutic landscape’ into physical, social and symbolic environments. Nevertheless, most healthcare settings, including those offering IC, continue to be grey and rather soulless, designed with ‘clinical efficiency’ in mind and not person-centeredness [17]. Furthermore, the designs reflect expert knowledge but involve little input from users [18].

More specifically, in IC the interventions are influenced by the patients, the staff and the environment, which in turn affect the individualisation and quality of patient outcomes [2, 11, 12]. According to Killington et al. [6], a participatory rehabilitative milieu like IC implies a ward configuration that facilitates optimum physical activity, social interaction and psychological responses. A systematic review showed that the physical environment is a critical component both physically and cognitively in providing person-centred care for older people in institutional care [19]. Pryor [20] underlined that the creation of a rehabilitative milieu implies the establishment of an environment that can enhance the rehabilitation process and the outcomes, and the external and internal design and location of buildings, including allocation, adequacy, usage, location of equipment and availability of space, must also be considered. The care environment thus embraces physical and psychosocial aspects and the importance of the work culture in which the care takes place [6]. However, rarely awareness has been given to user perspectives regarding the physical design and practice environment in IC to make it supportive and to help facilitate patient participation in person-centred care [21].

### Conceptual framework

Patient participation, the practices environment and person-centred care frame the research aims and findings of this study. In a recent concept analysis of patient participation in the context of IC services the authors identified five defining attributes. First of all, patient participation within geriatrics reflects a biopsychosocial approach to rehabilitation that understands the older person as a biological, psychological and social human being in context. Second, patient participation is considered as relational, it should be based on alliances and trust within a team approach. Third, patient participation implies information and knowledge exchange in order to make well considered health conditions. Forth, patient participation implies a mutual engagement in meaningful activities, physical as well as social. Finally, patient participation in IC reflects commitment and adjustment in organizational structures, it must be understood within the practice environment and culture of care [9]. The European framework of patient participation distinguish between three types of participation, that is choice, voice and co-production, a useful guide to understand this phenomenon in IC services. Having a “choice” refers to user involvement and the ability to make informed choices. “Voice” relates to the active involvement of the person in decision-making collaboratives. While “co-production” describes how the older person engage in partnerships in the delivery of their own rehabilitation process, participating in both physical and social activities [22].

The practice environment consists of physical aspects (e.g. building design), psychological aspects (such as staff attitude and culture of care), as well as social dimensions (physical and social activities), all considered as essential in optimising person-centred care. In this manuscript we emphasise the interaction between the person and the environment; the older person has a range of competencies, e.g. the physical, cognitive and social health, which are functional abilities, while the environment is defined in terms of pressure or demands [23].

A fit between the person’s competency and the environmental demands leads to a positive outcome, while in the opposite case, a mismatch may produce negative results [23]. Thus, in rehabilitation, when considering the person’s competence in relation to the environmental demands, there must be a balance between stimulating and supportive dimensions to promote patient participation [6]. In this study we understand patient participation within an empowering practice environment as fundamental to achieve person-centre care [8].

Person-centred care implies placing patients at the centre of their healthcare in a way that is respectful and sensitive to the patient’s preferences, values and needs [24]. To be able to embrace the multidimensional needs of frail elderly persons, IC should be holistic and emphasise the person’s meaningful life [10] and capabilities [25]. Accordingly, one size rehabilitation does not fit all; there must be space for individualisation, including in the environmental dimensions [17]. In a person-centred culture, there are several persons, including patients, relatives and staff, and it also includes the supposition that the professionals’ work environment is an important aspect of person-centred care [26].

### Research aim

More knowledge is needed regarding the practice environment’s influence on patient participation among older people in IC services [27]. Thus, the aim of the current study was to explore the perspectives of older patients, their relatives and staff regarding the practice environment’s influence on patient participation in IC services. More specifically we will examine: “What are the important environmental determinants that influence patient participation in the IC services according to the informants?”. An examination of these issues is important to furthering our understanding of the complexity of patient participation in IC services to contribute to person-centred care.

## Methods

A qualitative investigation using a constructivist approach was employed to explore the perspectives of older patients, their relatives and healthcare professionals regarding IC services. A constructivist approach implies an epistemological position in which knowledge is regarded as contextual; consequently, person-situation/environment interactions were analysed based on experiences [28]. This is the third publication based on data from a larger qualitative interview study exploring patient participation in IC services [2, 11].

### Study context

The Nursing Home Agency in Oslo is Norway’s largest organiser of short- and long-term services and is responsible for approximately 44 nursing homes. To meet demographic changes and to increase and to coordinate the competency in geriatric rehabilitation, in 2015, the city of Oslo synchronised its services into four large IC institutions. The present study was carried out in three of four IC institutions. With a total of 355 beds (see Table 1), these facilities serve 12 (of 15) municipal districts in the west, east and north of Oslo. The empirical data was collected in six wards, two at each institution (each with 20–30 beds). The reason for including three IC institutions and six wards was to explore different environmental settings [7]. The primary goal of IC is to help older people live at home for as long as possible by providing rehabilitation, treatment, assessment and palliative care, which is delivered by an interdisciplinary team. An initial family meeting is held within the first two to three days as a standard routine [12]. Most patients come from hospitals due to dependency in activities of daily living, but some are relocated from other IC units or admitted directly from their homes. Patients in IC typically receive medical treatment, home assessment, have the opportunity to participate in physical and social activities to manage activities related to daily living and receive follow-up services from the districts after being discharged to their homes.

**Table 1:** Facilities and participants’ descriptors

FACILITY	FACILITY A	FACILITY B	FACILITY C
Year of construction	1950/1951	2017 (1925)	1961 (2004)
Renovated	2007	–	2007
Beds/wards	122/5	96/4	137/6
Patients included			
Total	5	5	5
Sex (female)	4	3	3
Relatives included			
Total	4	4	4
Sex (female)	4	2	3
Staff included			
Total	6	6	6
Sex (female)	5	5	5

The three IC institutions included in this study were all large former long-term care facilities with several floors and long corridors. Facility A had the oldest design; the toilets were small and the institution did not have many rooms for storing equipment, making the corridors untidy/cluttered. Facility B was newly renovated with a modern white design. In comparison, Facility C had pictures, green plants and light-yellow walls, making it homelier.

### Study participants

To embrace the heterogeneity of the older population, the sample in the present study was purposive. In total, we included 15 patients, 12 relatives and 18 healthcare professionals.

The following inclusion criteria for patients were used: age over 65, transferred to IC from hospital or from home because of acute or chronic sickness or frailty, in need of help to carry out daily activities and seeking to be able to live at home. Patients with severe psychiatric conditions or an insufficient understanding of the Norwegian language were excluded.

The 12 relatives were included as they were stated by the participating patients as their closest or preferred next of kin. The staff were included to achieve diversity in terms of clinical experience, nationality and age. An overview of the participants and facilities is illustrated in Table 1. The characteristics of the participants are further detailed in the result section.

### Data collection

Between April 2017 and February 2018, individual, semi-structured, face-to-face interviews, were completed with each of the participants, which were recorded as MP3 sound files. Semi-structured interviews are considered a suitable data collection method for gaining insight into the participants’ understandings, preferences and experiences related to a phenomenon. This format allows the interviewer to pursue a number of less structured questions while simultaneously permitting the elaboration of topics raised by the interviewee during the process to initiate reflections and thick descriptions [29]. The interview guides used for interviewing the patients and relatives were developed for the larger study and are provided as Additional files 1 and 2. For the healthcare professional interview guide see Kvæl et al., 2019b, p. 923 [11]. The main topics in the patient interview guides of relevance for this study were the transition from hospital to IC, the initial family meeting, meal routines, daily activities and the discharge process. Within the relative interview guide the most important topics were those focusing on the practical part of the environmental care organisation in light of patient participation. Regarding interview guide used interviewing the professionals, the possible link between environment and participation were highlighted within the topics describing the participation of patients in daily care and when detailing the organisation of care and routines.

The patients were interviewed twice; first, they were interviewed in their rooms during their stay in the IC unit, and then they were interviewed in their homes 2–4 weeks after discharge. The relatives were interviewed 2–4 weeks after patient discharge; these interviews were conducted in their homes (*n* = 6), at the IC institutions (*n* = 2), at their workplaces (n = 2) or in a cafeteria (n = 2). The patients were initially informed about the project and asked to participate by the staff, and with their consent, their preferred relatives were contacted by the researcher (first author). Staff suitable for inclusion were informed about the study by the researcher and were asked to participate. None of the participants dropped out of the study.

A responsive interviewing style was employed for all interviews to build trust [30]. The interviews were conducted by the first author, a female PhD-student who has broad experience within the field of geriatric rehabilitation as a physical therapist. All the participants knew the researcher’s role and background, which was helpful in establishing rapport. The patient interviews lasted 20–60 min, while the relative interviews lasted 45–90 min. The healthcare professional interviews took place in each participant’s workplace but in a room outside of the ward, and they all lasted approximately one hour.

### Data analysis

The interview data were analysed using thematic analysis based on Braun and Clarke [31], a widely used approach for recognising, analysing and presenting themes across data (Table 2).

**Table 2:** Thematic analyses based on Braun and Clarke [31]

Phase	Description of the process
1. Familiarising yourself with the data	Transcribing data (if necessary), reading and re-reading the data, noting initial ideas.
2. Generating initial codes	Coding interesting features of the data in a systematic fashion across the entire data set, collating data relevant to each code.
3. Searching for themes	Collating codes into potential themes, gathering all data relevant to each potential theme.
4. Reviewing themes	Checking whether the themes work in relation to the coded extracts and the entire data set, generating a thematic “map” of the analysis.
5. Defining and naming themes	Ongoing analysis to refine the specifics of each theme, and the overall story the analysis tells; generating clear definitions and names for each theme.
6. Producing the report	The final opportunity for analysis. Selection of vivid, compelling extract examples, final analysis of selected extracts, connecting the results of the analysis to the research question and literature, producing a scholarly report of the analysis.

At first, the authors read the interview transcripts closely, searching for meaning and patterns while taking notes. Using HyperRESEARCH software, the data were coded and organised into meaningful groups by the first author. The codes were extracted from quotes about patient participation. Furthermore, the codes were organised in groups and sorted into themes. Among several main topics for interviewing, some have been reported in previous publications, types of patient participation and their potential empowering or disempowering effects (2), and how the IC team perform their work balancing between standardised routines, limited resources and the patient’s needs [11], the physical as well as the psychosocial environments, became prominent patterns across the three data sets influencing patient participation. Accordingly, this publication reports exclusively the data considering the impact of the practice environment. As an analytical strategy in this study we first regrouped what the informants said about the physical and psychosocial environment, based on their experiences, before identifying what they said about the various types of patient participation related to these environments. Table 3 provides an example of the coding procedure.

**Table 3:** Example of coding procedure

Quote about the practice environment	Codes	Initial theme
‘At the hospital, she just threw up. But then when she came here, she starts to eat from the first meal and enjoys…. What a change! I think coming here, it is very bright and nice here. Yes, it is much warmer. At the hospital, it was freezing cold, with brick walls and huge ceilings and lots of pipes. Thus, coming here with a warmer colour on the wall, and the staff here are very nice, she experienced a true welcoming atmosphere’ (Daughter, Facility A)	Light and colour Friendly staff To feel safe To feel welcome First impression Healing milieu	Homely environments

The sample size was guided by Malterud’s model of information power (data saturation), which rely on the study aim, the sample specificity, the utilisation of theory, the quality of the dialogue and the analytical strategy [32]. During data analysis we experienced sufficient information power. The conceptual framework was used as a lens for abstracting the data for further analysis, resulting in the emergence of three main themes. It is important to emphasize that the various types of patient participation are not mutually exclusive and will in the following be presented and discussed interchangeably related to the practice environment. The final results were further presented for validation in a stakeholder group consisting of two patient representatives, one previous relative and two nurses working in the IC services.

## Results

The analysis resulted in three interrelated themes: ‘location and access to physical facilities’, ‘symbolic expression of patients’ and professionals’ possibilities’ and ‘participating in meaningful activities’ (see Table 4). Together the themes illustrate how the physical and psychosocial environment, as well as the variation in how the same and different environment can lead to differences in participation, both in types of user involvement or “choice”, shared decision making as in “voice” and social participation such as engagement in physical/social activities. At the end of the result section, a summary is presented of recommendations based on important environmental determinants identified through the interviews (Table 5).

**Table 4:** Results of the analysis

Initial themes	Main themes
Homely environments	Location and access to physical facilities
Connection to outdoor facilities	
Environmental practicalities	
Access to inside facilities	
A welcoming atmosphere	Symbolic expression of patients’ and professionals’ possibilities
The meal as a social arena	
Empowerment of professionals	
Leadership to promote person-centred care	
Social and physical activities	Participating in meaningful activities
Ability for self-management	
The importance of family meetings	
Interdisciplinary tools in daily care	

### Characteristics of the participants

Of the 15 patients included in the study, 10 were women, and five were men. All were between 68 and 97 years old with an average age of 85,6 years. The mean duration of stay was 4 weeks. Furthermore, six had initial symptoms of cognitive decline, i.e. disorientation or memory loss, 13 required home help after discharge, and 14 used a walker for balance support. Three of the patients lived with their spouses, and the remainder lived alone. The relatives (nine women and three men) had an average age of 63,3 years. Three of the 15 patients included had no close relatives.

Ten of 12 relatives had higher education, seven were retired, four were still working and one was disabled. The relationships to the patients were as follows: daughters (*n* = 6), sons (*n* = 2), sister (*n* = 1), husband (n = 1), daughter-in-law (n = 1) and support person (n = 1). All 18 staff interviewed (15 women and three men) had been permanent employees for a minimum of 1 year. The average age was 43 years. The staff participants held various roles in the interdisciplinary IC team: doctors (3), nurses (3), nursing assistants (3), physical therapists (3), occupational therapists (3) and district coordinators (3). Most of the staff included in the study had extensive work experience with an average of 14 years.

### Location and access to physical facilities

The first theme, location and access to physical facilities, involves the implications of the architectural design to patient engagement as well as the actual availability to utilise existing opportunities. Overall, due to the patient frailty and dependency, patients and their relatives were thankful to be allocated a place in an IC facility before returning home from hospital. The services were highly appreciated and regarded as important to the promotion of a safe clinical pathway for older people to return home; however, patients generally experienced that the services were not designed in accordance with a person-centred rehabilitation principle with respect to the building design and a focus on the heterogeneous capabilities and preferences for patient participation. For example, although some patients favoured the long corridors as suitable for self-training other with less cognitive abilities had problems to orientate, especially in combination with a modern white and sterile design. They described these environmental features as hindrances to social participation. One family member said:

Environmentally…, I don’t think my father had any particular contact with the other patients, and so it was… I don’t know if it’s because the architecture is new, but it was incredibly sterile and difficult to orientate. The corridors are quite similar, and there wasn’t a picture on the wall inside his room, so we both experienced the physical environment as very impersonal (Daughter, Facility B).

According to the data, patients who were less physically and cognitively dependent were the most satisfied with the environment and the possibilities for social participation. The patients with a high degree of dependence described themselves as more restricted to a smaller space (common room or wing of the ward) due to the geography of the ward, thus having few choices and less access to social activities and training facilities. Accordingly, several informants suggested that facility designs should ensure that patients with reduced mobility are located closer to the social and physical rehabilitation facilities and that they should at least be able to enjoy an inspiring view through a window, especially if the staff do not have time to bring them regularly outside the ward. One patient recalled the experience negatively:

And this view, I can… it’s printed on my retina. That blue gable, sticking out there. I really want to know what’s behind it. It’s the one I’m watching. That’s my view as well as that tall brick house, yes [...] I don’t know what to do with life. I just sit here looking at this blue corner, those trees and that tall horrible house … I would rather see the sky … (Female patient, Facility C).

The patients described various needs in terms of capabilities and preferences for participation. Accordingly, while some were likely to engage in social activities, others wanted privacy and stress-free environments. Most participants in all three facilities highlighted their personal rooms, which had private bathrooms and offered the opportunity to withdraw, as a positive aspect of the environmental design: ‘Rehab is great. I have my own room. And I like privacy, being alone, with my crossword puzzles and reading’ (Female patient, Facility B). In addition, the patient rooms were considered space for social interaction with visitors and relatives; however, for some the patients’ rooms were also associated with passivity, which was the case for the large living rooms in the wards as well. The oldest patient stated: ‘Of course, you could sit in the living room, but I never saw anyone there...’ (Female patient, Facility A). Consequently, as underlined by the professionals themselves, most patients were likely to sit in the corridors as this location offered more opportunities for stimulation and interaction with healthcare professionals, who were more visible in the corridors.

Furthermore, the results suggest that patient participation in IC services, such as choice and patient engagement in activities, is highly associated with the physical allocation of room, determining the access to existing facilities also located outside the ward. For example, Facility B had training equipment and daily training programmes within the ward, which were perceived as very positive, while at Facility A and Facility C, the training facilities were located in the basement, resulting in less patient access and use of these facilities.

We need rooms that provide access to exercise—an exercise kitchen, a gym in the ward and places to have conversations with the patients…. And we also need high enough chairs so the patients can stand up and move independently in order to participate (Physical therapist, Facility A).

### Symbolic expression of patients’ and professionals’ possibilities

The second theme, ‘symbolic expression of patients’ and professionals’ possibilities’, implies that the practice environment represents social meaning shaped by reciprocity and interaction, which is best understood within the context in which they develop. Aspects of importance in all types of patients’ participation in care were related to the psychosocial dimensions of the facilities. Participants, as a sign of the importance of patient voice, emphasised the relevance of the facility projecting a welcoming atmosphere as a first impression. Five of the patients described their transitions to the IC as frustrating. Some reported that the taxi driver did not know which department to bring them to, resulting in long waits, and others said that no one in the IC unit knew they were coming. Since older people admitted to IC were considered a vulnerable patient group, a welcoming environment was understood as a symbol of control and safety, and thus an important environmental determinant to enable the patient voice.

That transition was unfortunate because it was in the afternoon. I think it was a Friday, so it was a temporary employee who did not know the routines and the building. She (their mother) was placed in the room, the bed stood next to the wall, and due to her right femur fracture, she had learned at the hospital that the bed should stand the other way. And the nurse said ‘that’s not possible’…, then she left and provided no more information. Then she called us and said ‘I’m in a basement. I don’t know why I’m here. I’m stuck’ (Son, Facility B).

Furthermore, the meal situations were highlighted as crucial for patient participation, both as a social arena as well as the relevance of healthy food to promote physical recovery and further engagement. The relatives underlined the symbolic role of nutrition in geriatric rehabilitation, the relevance of monitoring nutritional status as a foundation for patient recovery in IC. One relative was concerned as the main goal of her mother in law was improved nutritional status:

It does not seem that they realized that she hardly ate. When relatives express concern, they should at least know what she has eaten, and measure what she has drunk, I think you can expect that. I understand that they have a lot to do, but wonder if they see the importance of nutrition in rehabilitation (Daughter in law, Facility A).

The patients also emphasised the importance of delicious food and that the manner of serving meals signalled in what way the facility values them and takes their preferences into account. Although a few of the patients described the meals as satisfying organized in small groups, the food was in general described by patients and relatives as mass production. Hence, the aspects of mealtimes where they felt that the staff expressed limited time towards them, and there were periodically few fellow patients to communicate with (due to frailty or the organisation of large tables and who sits together), were largely described in a negative and non-participating manner. This was particularly the case at Facilities B and C.

For lunch yesterday, we got served on a plate, a bit dry ... I call it a fish cake and some cooked vegetables. I think that was a poor serving. That’s my personal opinion. I think it seemed a little petty. I mean ... if they had put on a potato and some sauce, that plate would have looked completely different, plus that fruit. It had made a huge difference! As well as to put the plate in front of the patient instead of just pushing it across the table. It’s about dignity, to feel that you are a human being (Male patient, Facility B)

Furthermore, most patients reported that they were afraid of being a burden, which might be another factor related to interactions between patients and the practice environment. When the staff members expressed that they had limited time, patients felt disinclined to ask for help, a crucial environmental determinant influencing the patients’ voices. Indeed, as highlighted by the participants, the meal situation can be understood as a symbol of dignity.

Several staff members described that frequent reorganisations in the healthcare system caused frustration among workers in IC as well as among the municipal district coordinators, reducing their professional room and time to engage with the patients in the rehabilitation process. Most of the professionals experienced the changes as a symbolic expression of the environment, described as ‘orders from above’ and not as processes for their or the patients’ influence. They underlined that inappropriate organisation and utilisation of existing resources indirectly symbolised an environmental barrier to all types of patient participation. The oldest staff member, an occupational therapist with extensive years in the field, stated:

The economy is often a barrier to what we want. In addition, we do not experience being heard regarding how to organise our profession in the best way for the patients. We (the therapists) are assigned to different wards to support staff, but we also need our own professional development. We need a leader who sees us and who can help us prioritise, a professional direction (Occupational therapist, Facility C).

The staff members indicated that they desired to be appreciated at work and that leadership had a role to play in mentoring and in establishing a person-centred culture emphasising patient participation. In their daily work with patients and their relatives, some experienced that the freedom of discretion in a person-centred practice environment was hindered by an autocratic leadership style; however, there were also examples of good leadership experiences promoting flexibility and empowerment of staff. One of the nurses said:

It was a departmental meeting where they discussed a very poor statistic, where we had not documented properly. But, I have never left a departmental meeting with such a positive experience, so there is something about how they convey things (Nurse, Facility A).

### Participating in meaningful activities

The third theme, ‘participating in meaningful activities’, embraces the fact that stimulating rehabilitative activities, physical as well as social, must be offered in an environment in which the professional knowledge and overall philosophy of care plays an important role. One of the most striking issues addressed by all the participants was the highly sedentary IC lifestyles. Both patients, relatives and healthcare professionals working in the three facilities described limited opportunities for inside and outside activities offered by the facilities, as well as a lack of a rehabilitative or person-centred thinking. Family meetings during which the patients and their relatives could express their voices and needs were highlighted as positive.

Other than meals and occasional training with therapists, patients experienced little to do in the wards. Although the staff were described as kind and service-oriented, little occurred in the IC units to stimulate the patients, and this was a common pattern across the data. While staff members attributed this to a lack of time and resources, saying they were forced to prioritise practical tasks at the expense of spending time with the patients, several patients described the routinised care as boring and passive. Furthermore, relatives noted the lack of a person-centred practice environment in which staff were able to perceive the human beings behind the diagnoses. In fact, they observed that the staff were doing activities ‘for’ instead of ‘with’ the patient, a model of care not in accordance with a rehabilitative philosophy.

My father is a bit lazy and not very practical. He said to us when we visited him there, ‘I just lie down on the bed. Then, someone comes and dresses or undresses me… I’ve outsourced these services’. So, he was never ‘pushed’ in a way, or held accountable. They never succeeded in finding his motivation (Daughter, Facility C).

In addition to describing limited resources, participants highlighted that this lack of a rehabilitative or person-centred thinking was also a result of an impractical approach to how the staff completed duties. For example, one of the patients suggested that instead of constantly asking for hot water for tea, it could have been made available on a trolley in the corridor. A relative recommended access to small dishes of yoghurt and fresh fruit in a location that they could access themselves. Similarly, a staff member suggested that patients be engaged in cooking. Indeed, they all emphasised that there were small practical things to be done to make the environment more participatory and stimulating.

An outdoor garden and access to a cafeteria were highlighted as important environmental determinants for meeting relatives, grandchildren and pets. In Facility C, the outdoor garden was described as healing and very nice by the patients and relatives; however, the cafeteria was closed during the time of the study, which was described as unfortunate, framing such spaces as indicators of a person-centred practice environment. When asking what could change to produce a more participatory environment, one patient said:

Homemade food and a place to buy a newspaper, a sandwich, anything. I believe those are two of the most important things because then you have the opportunity to leave the room, move your body and socialize (Female patient, Facility C).

Another aspect that patients and relatives greatly appreciated in all the facilities was the arrangement of family meetings with staff or care teams, which was described as important to achieving shared decision making. Within these meetings the patients and their relatives experienced the opportunity to voice their needs and goals as the healthcare staff asked the question ‘What is important to you?’ The healthcare professionals on the other hand felt that asking this question routinely was helpful to develop a safe and accurate care plan for the patient. In fact, the family meetings were described as a success criterion in order to promote patient participation and to discuss the length of stay and post-discharge follow-up services.

I think the family meeting was very nice and useful. You know, I experienced getting such good information and ... she, from the municipal district, and everyone else… they were really helpful and understanding (Female patient, Facility A).

The staff also highlighted their daily meetings around the blackboards as crucial to enable person-centred care. In every ward office they described a large blackboard hanging on the wall containing relevant information about the patients, such as name, age, date of arrival, diagnoses, patients’ goals, family meetings, fall history, pressure ulcer, urinary tract infection and other aspects, to promote patient safety, participation and interdisciplinary collaboration. The blackboard meetings, during which a brief review of each of the patients, an assessment of risk factors and status of progress, are provided, were experienced as an environmental determinant that influenced the professional way of working and further patient participation.

I think daily blackboard meetings promote a person-centred practice environment. We get to know the patient well. Yes, we place our honour in the multidisciplinary team around the patient. And when the ward is organised the best possible way, we also practice this way of thinking in daily patient care (Nurse, Facility B).

### Environmental recommendations

The environmental recommendations in Table 5 incorporate adaptations and modifications to the practice environment as well as advice for the individuals’ behaviours and arrangements.

**Table 5:** Environmental determinants influencing patient participation based on the interviews

A warm colour on the walls, pictures and green plants that make the place homelier	
The importance of the window view when allocating sleeping accommodations	
Locate the less mobile patients closer to the social facilities	
Therapy gym within or close to the ward available for the patients (and access to garden)	
A welcoming atmosphere and first impression as a symbol of control and safety	
Homemade and healthy food served in a dignified way as a social arena	
Empowerment of professionals to obtain job satisfaction and competent employees	
Recognition and training of leadership to promote a person-centred practice environment	
Organise daily social activities to stimulate the patients physically and cognitively	
Access to kitchen, small diches, fresh fruit and hot water to supply themselves	
Family meetings as a standardised routine to voice the patients’ and relatives’ needs	
Blackboard meetings every morning as an interdisciplinary tool for continuity	

## Discussion

To the authors’ knowledge, this is the first study to report exclusively on patients’, staff members’ and relatives’ views on important physical as well as psychosocial environmental determinants that influence their participation in IC services. Patients, their relatives and professionals all stated that the practice environments influenced patients’ participation in IC. As stated, patient participation in IC might take several forms; to make informed choices through user involvement, to voice their goals and needs in shared decisions making, as well as to engage in physical and social activities as co-producers of the rehabilitation process. According to the participants, ensuring that the environment has positive, empowering impacts on the formation of alliances that support tailored information and informed choices is important. Furthermore, the environment should ensure patient engagement in meaningful daily activities and invite patients and their relatives to voice their needs into shared decision making, such as family meetings. To focus this discussion, the following three topics related to the practice environment are first discussed: 1) a core component of quality healthcare, 2) support of individual capabilities and the signal of patient value and 3) facilitators of shared decision making and effective staff-patient relationships. Then, some of the strengths and limitations of the study are presented before outlining the practical implications of the study.

### A core component of quality healthcare

The findings of the current study correspond with those of Colley et al. [33], who stated that growing research demonstrates the potential for healthcare environments to support recovery and participation following illness or injury, impacting a range of outcomes. They referred to the Internal Classification of Functioning, Disability and Health (ICF) developed by the WHO, which identifies environmental aspects as a core component of quality healthcare [33].

Vik et al. [3] suggested that interactions between individuals and their environments are dictated by the environment’s influences on the individual’s participation. These authors’ findings are in line with our own: the environment is a function of multidimensional elements that are exterior to the patient, such as physical, social and societal features of environments. All these features are referred to in our findings and seem to be of great importance for the various types of patient participation in a person-centred practice environment [3].

Regarding the core component of quality care, Ulrich [34] highlighted three key considerations for creating less stressful environments that are more conducive to health and well-being: (1) the environment must provide a sense of control and access to privacy; (2) it must include social support; and (3) it must provide access to nature and other positive distractions. Our findings support all these aspects as crucial for patient participation. For example, the ward configuration should be homely and easy to orientate as well as should offer private rooms with the opportunity to withdraw. Furthermore, to include social support, the staff must be visible, and the wards should organise activities to stimulate the patients both physically and cognitively. Finally, access to an outdoor garden, gym facilities and a cafeteria were found to be positive distractions facilitating patient participation.

### Support of individual capabilities and the signal of patient value

In accordance with Lawton and Nahemow, the participants described the importance of an environment that was easy to navigate, facilitated interactions with other patients and fostered opportunities to watch the sky and trees. In optimal person-centred settings, all participants noted, the environment should accommodate for losses of physical function in people, such as impaired vision and mobility [23]. Remarkably, this was not the case in this study, even after a major renovation, such as in Facility B; however, the findings are in line with Nordin et al. [27]. Furthermore, Lawton and Nahemow [23] identified the necessity of being in practice environments that recognise the scale of functioning, from dependence in daily activities to independence, and the participants supported this. The participants’ experiences were in accordance with Lawton and Nahemow’s view that the central goal of healthcare is to harmonise the degree of support given by the environment to the degree of capabilities of the person; however, the participants stated that only small and affordable changes were required, and many of these were related to the professionals’ experience in fulfilling patients’ needs. Like Moore et al. [35], the staff realised that the practice environment was not conducive to a person-centred practice and that staff missed opportunities to develop professionally.

The central principle for participation is the relationship between the individual abilities and the demands and resources available in the environment. As people age and their competences decline, environmental demands increase, resulting in a need to compensate or adapt to new conditions to avoid negative outcomes [36, 37]. Competence involves human possibilities, such as intelligence, sensory skills, motor skills, etc., while the environment implies demands, meaning physically as well as psychosocial aspects, such as stress and expectations [38]. Hence, the findings also suggest that the environment symbolises social meaning influencing participation. For example, a welcoming atmosphere could be understood as a symbol of safety, while a nicely prepared meal might be a symbol of dignity, thus the environment conveys symbolic meanings of caring and uncaring for the older person. A Swedish study illustrated that when new patients are met by the staff, shown around and receive sufficient information, this is perceived as a symbol of control, creating a good first impression and a feeling of safety and being welcome. Furthermore, the same study demonstrated that non-institutional smells, such as food, bakery goods and coffee, could have an impact on recognising oneself in the environment and in promoting the feelings of homelikeness [39].

### Facilitators of shared decision-making and effective staff-patient relationships

Patients, relatives and healthcare professionals reported that the environment does not sufficiently facilitate shared decision making, power sharing, effective staff-patient relationships or holistic health care, which are important in person-centred environments [2, 11, 12]. Person-centred processes focus on providing care through a scale of activities to secure a healthy culture in which patient participation is considered a core strategy [17]. However, this is not something the participants experienced. The participants viewed the economy and access to facilities as barriers to meeting the older patients’ need for participation. The informants highlighted that the stay in IC was associated with a passive existence. They wanted a friendlier environment that encouraged participation in activities to enhance their well-being. Furthermore, they wanted improved mobility options, enhanced social networking opportunities and opportunities to engage in everyday activities. The findings are highly in accordance with Killington et al. [6], who concluded that patients in rehabilitation should be offered choices and access to both inside and outside facilities. This means that limiting rehabilitation only to sleeping accommodations and therapy spaces cannot support best practice rehabilitation. Clearly, and supported by the results, the quality of time spent between therapy sessions, such as meals and access to meaningful activities in supportive facilities, should become a higher priority in designing person-centred IC facilities [6]. The healthcare professionals in the current study attributed the lack of patient participation in a person-centred practice to a lack of time and resources. This is line with Sjögren et al. [26], who concluded that an optimistic and supportive psychosocial atmosphere and a work environment where healthcare professionals face a balance between control and demands in their daily work to facilitate a person-centred care, seem to be of great relevance for leaders and managers in IC services for older persons. As underlined by McCormack and McCane, person-centred care is fostered in a healthful and participative culture [17].

Organising family meetings was appreciated by all the participants and was regarded as a way to facilitate patient participation and person-centred care. The meetings have the potential to highlight the patient competencies in light of the environmental demands, finding the balance between support and stimulating care. These findings are supported by the research literature [12, 40]. In a person-centred environment, the patient’s history must be known [2, 17, 38]. Consequently, the professionals experienced the blackboard meetings as helpful, as they made the patients’ competence visible through their medical history, goals, preferences and physical function. The main aim of IC services is to help patients live at home for as long as possible, and the services should therefore facilitate all types of participation and promote adequate behaviour in the balance between environmental demands and individual competency. There is considerable evidence in the literature that older people should actively participate in order to shape their environments [41]. For example, the environmental proactivity hypothesis states that older persons have the potential to change their environments in a proactive way to meet their needs and to stay independent [42]. Similarly, Ouwehand et al. [43] claimed that older persons do not solely deal with decline, but they also proceed to evolve as humans and to fight for personal development by shaping environments that facilitate success. The practice environments, including the physical, psychological and social dimensions, have remarkable influence on the various types of patient participation in all groups of age, particularly older persons highly dependent on their local facilities for support and assistance. Older people may be especially vulnerable to changes in these immediate environments, in light of their necessity and importance to preserve the person’s identity [44]. Consequently, the practice environment is a core element in intermediate healthcare services and should, as highlighted by the findings, be able to balance the individual patient’s competences with the environmental demands to support patient participation in a person-centred manner.

### Strengths and limitations

To secure the trustworthiness of data in qualitative research design it is of great relevance to include methodological strategies [45]. To the authors’ knowledge, this is the first study to focus exclusively on patients’, healthcare professional members’ and relatives’ views on the important environmental determinants that influence patient participation in IC services.

The trustworthiness of the study is strengthened through the use of well-known conceptual frameworks, the fact that two authors were involved in the analysis, the use of quotes in the results section and previous research presented in the discussion to substantiate the findings. In addition, three different data sources were used to ensure that the data collected were rich, robust, comprehensive and well-developed [46]. The manuscript follows the consolidated criteria for reporting qualitative research (COREQ).

The pre-understanding of the authors was that the various types of patient participation in the context of IC services are influenced by the practice environment. Both authors have background in physiotherapy and long clinical and/or research involvement within geriatric rehabilitation and healthcare services for older people. The first author (PhD-student) conducted all the interviews, and the second author (Professor, PhD) served as a discussion partner to balance a close with an analytical distance to the data material [47]. In addition, field notes served as a reflexive tool (memo) to challenge taken for granted assumptions. Overall, the process of analysis was attempted to be transparent and to be presented with clarity [48]. Transcript were not returned to participants; however, the researcher always summarised the interviewee’s argument in order to make sure he or she was understood correctly. Although we have discussed our findings in a stakeholder group, consisting of patients, relatives and staff, including a reference group from the beginning to the end would have been a strength [49]. A limitation could be that our findings essential to the three urban IC institutions included are not necessarily transferable to institutions in more rural districts or other countries [50]. In addition, although we believe that the purposive sampling reflects the heterogeneity of the IC services, all the patients were Norwegian because they had to speak the language fluently. Accordingly, any culturally sensitive differences in light of nationality were not explored within the material. Nevertheless, we hope our findings will widen and nuance the understanding of environmental influences in IC services for older people.

### Practice implications

This research provides the patients’, relatives’ and healthcare professionals’ perspectives of the necessary requirements to consider when designing IC services. The environmental determinants and recommendations based on the interviews are summarised in Table 5. Apart from the architectural design, the participants emphasised small, practical changes to make the environment more participatory and person-centred. The present study contributes important knowledge about the complex environmental determinants that might be exterior to the patient, for example the physical, psychological and societal features of environments, as well as how they must be balanced in accordance with the older persons’ internal competencies to optimise all types of patient participation in a person-centred IC culture.

## Conclusions

Overall, the participants generally experienced the IC services as not designed in accordance with the person-centred rehabilitation principles with respect to building design and a focus on the heterogeneous capabilities and preferences for patient participation. To optimise rehabilitation in IC for older people, the ward configuration could focus on supportive environments that facilitate patient participation and provide options for the patients and relatives to independently access the facilities. Furthermore, it seems important to be aware that the rehabilitation environment conveys symbolic meanings of caring and uncaring for the older person expressed through the way of care. The study highlights the relevance of the older patients being able to escape the clinical environment to socialise with relatives and other patients. This knowledge might be of relevance to staff, managers, building planners, architects and policy makers to optimise person-centred care in IC services. To foster patient participation, the practice environment should align more with the model of person-centred rehabilitation, which also includes the work environment of the healthcare professionals.

## Supplementary Information

**Additional file 1.**

**Additional file 2.**

## Data Availability

As the data collection approval for the main study states the data to be available only to the researchers, data and materials collected for this manuscript will not be shared.

## Abbreviation

IC: Intermediate care
