# Supplementary material for: The practice environment’s influence on patient participation in intermediate healthcare services – the perspectives of patients, relatives and healthcare professionals
Source: BMC Health Serv Res. 2021 Feb 25;21:180. doi: 10.1186/s12913-021-06175-z (PMC7908719; doi:10.1186/s12913-021-06175-z)
Supplement: Supplementary file 1 — Additional file 1. [file 12913_2021_6175_MOESM1_ESM.docx]

**Interview Guide for Patients—First Conversation**

The first conversation interview occurs at the intermediate care (IC) institution at a convenient time for the patient. The interview takes place in a room where the researcher and patient can talk undisturbed and the conversation can be recorded. This first conversation is based on the patient’s transfer experience from the hospital to IC, the initial family meeting, the rehabilitation and the patient’s overall experience so far regarding patient participation. The interview will be relatively open, but the following thematic areas will be highlighted. The sub-questions serve as a checklist for the interviewer to ask the patient, in case the participant does not cover these areas throughout the conversation.

1. **Participation in the hospital to IC transition**

- Please describe your experience on the day of transition?
- Please describe important factors regarding participation in a discharge process?
- Please describe who made the decision regarding transition to an IC unit?
- Please describe the information regarding transition to an IC unit provided by staff at the hospital?
- Please describe your experience regarding reception at the IC unit?
- Please describe your experience with the information provided at the IC unit?
- Please describe what a good reception looks like?

1. **Participation in the initial family meeting**

- Please describe the atmosphere at the family meeting?
- Please describe your opportunity to participate?
  - Did the staff ask you what matters to you?
  - Were they interested in your points of view? Please provide examples.
  - To what extent do you want to participate and when?
- Please describe your experience with the follow-up of your shared views?
- Please describe your experience with the involvement of relatives?
- Please describe your experience with the establishment of goals and plans for your IC stay?
- Please describe your thoughts regarding patient participation in general?
- Please describe your thoughts regarding what staff may learn from you?

1. **Practice environment and the rehabilitation so far**

- Please describe the treatment usefulness so far?
- Please describe your experience of safety and of “being seen” as a person?
- Please describe your thoughts regarding the facilities (physical and social)?
- Please describe your thoughts regarding the care organisation?
- Please describe the staff support regarding your personal goals?
- Please describe what you associate with good rehabilitation?
- Please describe patient opportunities for participation at IC? In daily care, treatment, decision-making, in social activities.
- Please describe if your stay meets your expectations so far? Do you have any examples?

Open-ended questions are asked first, but the researcher can also ask about situation descriptions given by other participants similar to what the patient is describing. Such input is intended to clarify what the patient describes by commenting on other people's experiences. Thus, the patient can agree or disagree with others’ opinions. At the interview’s end, the researcher will sum up and check if participants were correctly understood. The researcher will open up the conversation for any additional information by asking, "Is there anything I haven't asked about that you feel may be of importance?"

**Interview Guide for Patient—Second Conversation**

The second conversation interview occurs in the patient's home, at the IC institution or wherever the patient prefers. The interview takes place in a room where the researcher and patient can talk undisturbed and the conversation can be recorded. For the most part, this conversation will be based on the discharge process from IC to home, the experience of coming home and the overall experience of being an IC patient. The interview will be relatively open, but the following thematic areas will be highlighted. The sub-questions serve as a checklist for the interviewer to ask the patient, in case the participant does not cover these areas throughout the conversation.

1. **The discharge process from IC to home**

- Please describe what you thought about your IC length of stay?
- Please describe your thoughts and feelings on your day of discharge to home?
- Please describe your thoughts regarding the information provided by staff in advance of your discharge? The quality of the information, about follow-up services, time to prepare.
- Please describe who decided when your discharge to home should take place?
- Please explain factors important to patients in a discharge process?
- Please describe your trust in the overall system (hospital/IC/district)? Please provide examples.
- Please describe your experience with the overall information quality provided (hospital/IC/district)? Please provide examples.

1. **The home situation after discharge**

- Please describe your experience of coming home?
- Please describe the information provided by staff regarding further opportunities for participation in social or physical activities in the district?
- Please describe what you believe are success factors for a good/safe transition from IC to home?
- Please describe the quality of home help you receive today?
- Please describe how much your IC treatment has helped with your return to home?
- Please describe your feelings of safety today?

1. **The overall experience of patient participation and of being an IC patient**

- Please describe a day in IC? Daily activities, access to facilities, design, staff availability etc.
- Please describe your experience of the overall treatment you received?
  - Are patients seen as persons?
  - Does the staff have enough competence?
- Please describe your thoughts regarding what you perceive as “good” treatment?
- Please describe your opportunity for treatment involvement?
- Please describe your opportunity for shared decision-making?
- Please explain your preference for participation in care, in social activities, in daily activities, in the meal situation? Do you have any examples?
- Please describe your recommendations to improve patient IC participation?
- Please describe whether you would recommend an IC stay to other patients?

Open-ended questions are asked first, but the researcher can also ask about situation descriptions given by other participants similar to what the patient is describing. Such input is intended to clarify what the patient describes by commenting on other people's experiences. Thus, the patient can agree or disagree with others’ opinions. At the interview’s end, the researcher will sum up and check if participants were correctly understood. The researcher will open up the conversation for any additional information by asking, "Is there anything I haven't asked about that you feel may be of importance?"
