# Supplementary material for: The practice environment’s influence on patient participation in intermediate healthcare services – the perspectives of patients, relatives and healthcare professionals
Source: BMC Health Serv Res. 2021 Feb 25;21:180. doi: 10.1186/s12913-021-06175-z (PMC7908719; doi:10.1186/s12913-021-06175-z)
Supplement: Supplementary file 2 — Additional file 2. [file 12913_2021_6175_MOESM2_ESM.docx]

**Interview Guide for Relatives**

The interview occurs at the IC institution or elsewhere at the participant's request. The interview takes place in a room where the researcher and participant can talk undisturbed and the conversation can be recorded. Participants are initially informed about the project and then provide their written consent. The interview will be relatively open, but the following thematic areas will be highlighted. The sub-questions serve as a checklist for the interviewer to ask the participant, in case the participant does not cover these areas throughout the conversation.

- **Your role as a relative in IC**
- Please describe your role as a relative in intermediate healthcare services?
- Please describe your relationship to the IC healthcare professionals?
- Please describe how much you were involved in determining the care plan on behalf of your relative?
- Please describe the quality of care you feel your relative received at IC?
- **Your experience as a relative in IC services**
- Please describe how you (as a relative) experienced the discharge process from the hospital to IC? (e.g. information, planning, decision-making participation)?
- Please describe your view of how your relative was treated by staff upon IC arrival?
- Please describe whether you feel your relative was listened to throughout the IC process (e.g. the initial family meeting, treatment and/or meal situations)? Please explain and provide examples.
- Please describe whether you feel patients are "seen" at IC?
- Please describe whether you feel the information at IC is clear and sufficient? Daily routines, what happens and when?
- Please describe your experiences (as a relative) of having your comments taken seriously, listened to and followed up on by IC healthcare professionals?
- **The meaning of patient participation in healthcare**
- Please describe your understanding of the “patient participation” principle?
- Please describe your opinion regarding older patients being involved in their care?
- Please explain to what extent you think older people should participate in their own rehabilitation?
- Please describe how older people should be involved in their own care? Please provide examples.
- **The environment and care organisation in light of patient participation**
- Please describe your opinion regarding organisation of care, resources, routines, facilities, procedures, design, etc.? Please provide examples on a general or personal basis.
- Please describe what factors are important to facilitate patient participation?
- Please describe what factors function as barriers to patient participation in IC services?
- Please describe your experience with how patient participation is supported by the management?
- Please describe your experience with how patient participation is supported across service levels?
- Please describe your opinion of IC as an offering to older patients along the clinical treatment pathway?

Open-ended questions are asked first, but the researcher can also ask about situation descriptions given by other participants similar to what the participant is describing. Such input is intended to clarify what the participant describes by commenting on other people's experiences. Thus, the participant can agree or disagree with others’ opinions. At the interview’s end, the researcher will sum up and check if participants were correctly understood. The researcher will open up the conversation for any additional information by asking, "Is there anything I haven't asked about that you feel may be of importance?"
